# Osteopoikilosis: a case report

**DOI:** 10.1186/s13256-023-04025-6

**Published:** 2023-07-12

**Authors:** Behzad Mohsenpour, Amjad Ahmadi

**Affiliations:** 1 Department of Infectious Diseases, Faculty of Medicine, Kurdistan University of Medical Sciences, Sanandaj, Iran; 2 Department of Microbiology, School of Medicine, Hamadan University of Medical Sciences, Hamadan, Iran; 3 Zoonoses Research Center, Research Institute for Health Development, Kurdistan University of Medical Sciences, Sanandaj, Iran

**Keywords:** Osteopoikilosis, Buschke–Ollendorff syndrome, Osteopecilia

## Abstract

**Background:**

Osteopoikilosis, also referred to as disseminated condensing osteopathy, spotted bone disease, or osteopecilia, is a rare bone disorder. The case presented here showcases multiple disc lesions in the spine, extensive multifocal skin lesions, and positive test results for dermatomyositis and multifocal enthesopathy, accompanied by neurological symptoms. This manifestation represents a novel variant of the disease.

**Case presentation:**

Our patient is a 46-year-old mosque Kurdish servant presenting with complaints of pain in the right leg, lower back, right hand, and neck. Additionally, the patient has been experiencing redness in the right buttock and ipsilateral thigh, as well as gradually expanding and stiffening skin lesions on the left shin for the past 3 weeks. Painful neck movements and a positive Lasegue test were also observed in the right leg. The patient reports pain in the right buttock accompanied by a substantial erythematous area with induration measuring 8 × 15 cm, as well as an erythematous and maculopapular lesion measuring 6 × 18 cm on the left shin.

**Conclusions:**

Our patient is a 46-year-old man presenting with complaints of skin lesions and pain in the lower back, pelvis, neck, and limbs. The X-ray reveals shoulder, pelvis, knee, and ankle involvement, while spinal involvement is observed in the neck and lumbar region. Furthermore, the bone scan indicates extensive enthesopathy in various regions, a unique manifestation not previously reported in similar cases.

## Introduction

Osteopoikilosis is a rare bone disease initially described by Albers-Schonberg in 1915 [1]. It has an estimated prevalence of 1 per 50,000 individuals and is classified as an autosomal dominant osteosclerotic (ADO) dysplasia characterized by a distinct radiological appearance [2]. This condition is also called disseminated condensing osteopathy, spotted bone disease, and osteopecilia [3]. There are three forms of the disease: speckled type, striated type, and mixed type [3]. Autosomal dominant osteopoikilosis is associated with heterogeneous mutations in the LEMD3 gene [4]. The primary pathology of this condition involves disruptions in the bone maturation process, classifying it as a form of bone dysplasia [5]. The Buschke–Ollendorff syndrome is characterized by concurrent skin involvement, with skin biopsies and pathological examinations revealing an abundance of fibroelastins in the middle and deep dermis [6]. Rare complications associated with this syndrome include spinal canal stenosis, dacryocystitis, and malignant transformation [7]. Approximately 25% of Buschke–Ollendorff syndrome cases exhibit dermatofibrosis lenticularis disseminata, which exhibits colloid formation and resembles scleroderma. This manifestation is attributed to the proliferation of activated fibroblasts and may occasionally result in palmar-plantar keratoderma [2, 3, 8]. Bone densitometry typically shows normal results [9]. The condition is characterized by the presence of elastic connective tissue in this diffuse syndrome [10]. The current case involves multiple disc lesions in the spine, extensive multifocal skin lesions, positive findings for dermatomyositis and multifocal enthesopathy, and accompanying neurological symptoms. This presentation represents a novel variant of the disease.

## Case report

The patient is a 46-year-old Kurdish male mosque servant presenting with pain in the right leg, lower back, right hand, and neck. The patient reported experiencing symptoms of redness in the right buttock and ipsilateral thigh, as well as skin lesions in the left shin, which started gradually, expanded, and became stiff over the course of 3 weeks. Additionally, the patient complained of pain in the lower back and pelvis that worsened with walking, significantly affecting his ability to walk normally. Two years prior, the patient underwent surgery for cervical disc herniation and was subsequently treated with acetaminophen and chlordiazepoxide but experienced no improvement in symptoms. The patient had a history of smoking 20 packs of cigarettes per year but quit smoking 8 years ago. At the time of the study, the patient admitted to using a small amount of opium. During the examination, the patient’s vital signs were stable, and a surgical scar from the disc surgery was noted on his neck. The patient reported painful neck movements, and the Lasegue test of the right leg yielded a positive result. He also experienced pain in the right buttock, and an area of erythema with firm induration measuring 8 × 15 cm was observed. Furthermore, an erythematous and maculopapular lesion measuring 6 × 18 cm was present on the left shin. The right proximal muscle strength was assessed as 4.5, as was the strength of the right lower limb. Deep tendon reflexes (DTR) in the right upper limb were + 3, while in the Achilles lower limb, they were + 4 with clonus.

Preoperative Magnetic resonance imaging (MRI) of the neck revealed the following findings: Cervical lordosis with spondylolysis changes, posterior bulging disk in C3–C4, C4–C5, and C5–C6, resulting in compression of the thecal sac at the affected levels. Additionally, there was evidence of myelomalacia in the cervical cord at the C4 level. Furthermore, a hemangioma was observed in the T1 vertebral body.

Pelvic MRI: Multiple enumerable low signal foci in bilateral femoral head compatible with benign sclerotic lesions such as osteopoikilosis.

Brain MRI: Normal.

Back MRI: Decreased lordosis and dehydration, bulging in L4–L5 level, and bared base seen in L4–L5 and central L5–S1.

Triphsic whole body scan: Enteropathy involving the upper portion of right SI joint, left anterior superior iliac spine, right pelvic brim, bilateral patella, right tibia tuberosity, and bilateral medial malleoli.

Main symptom: Pain in limbs, neck, and lumbar spine, and skin lesion as erythema and warmness in the upper tie, buttock, and leg.

Medical, family, and psychological history: Surgery for disk herniation in the neck spine, diabetes mellitus in his mother and mild hypertension in his father, a history of cigarette smoking (18 packs per year), a history of inhalational opioid use, and no history of psychological disorders.
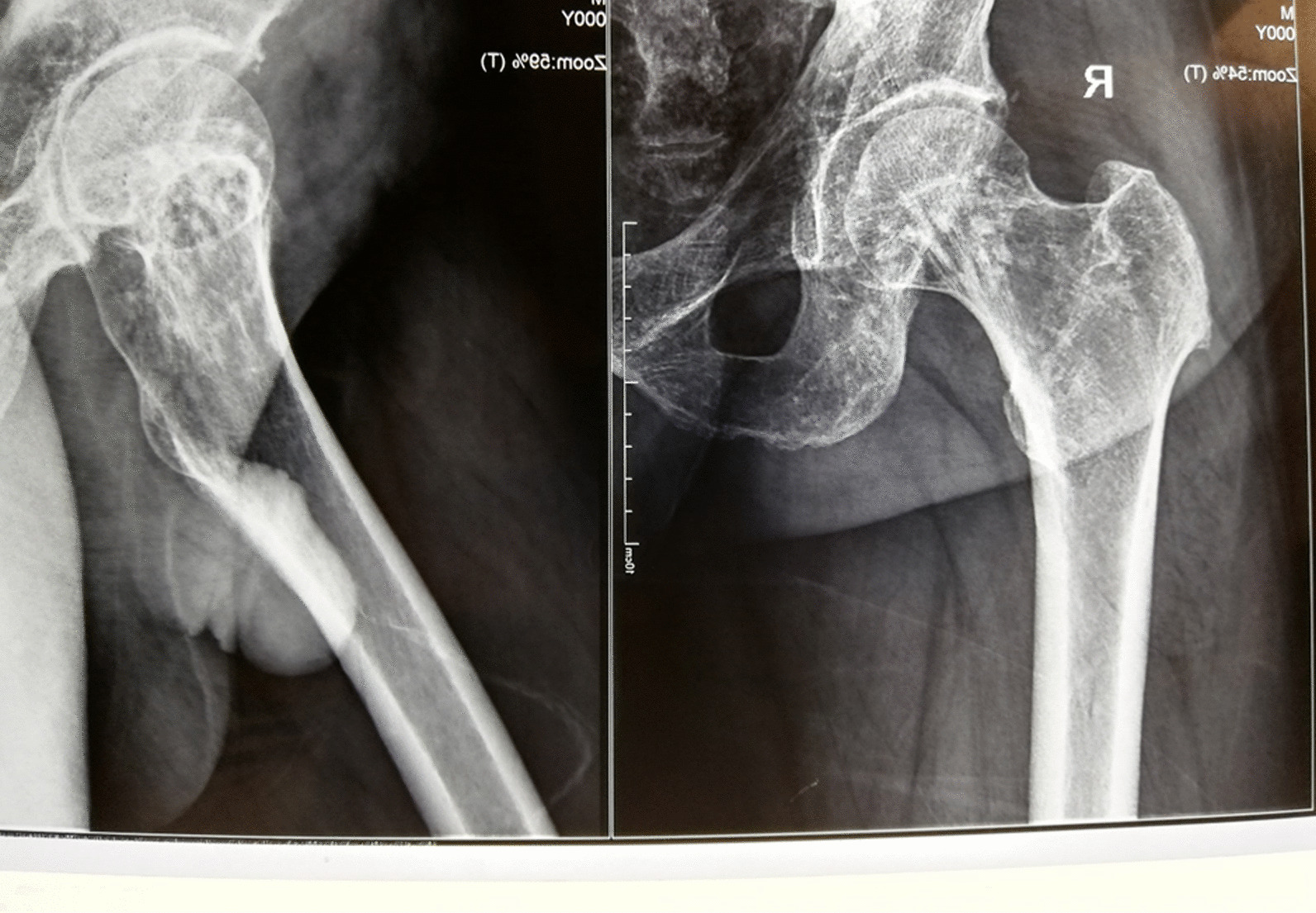



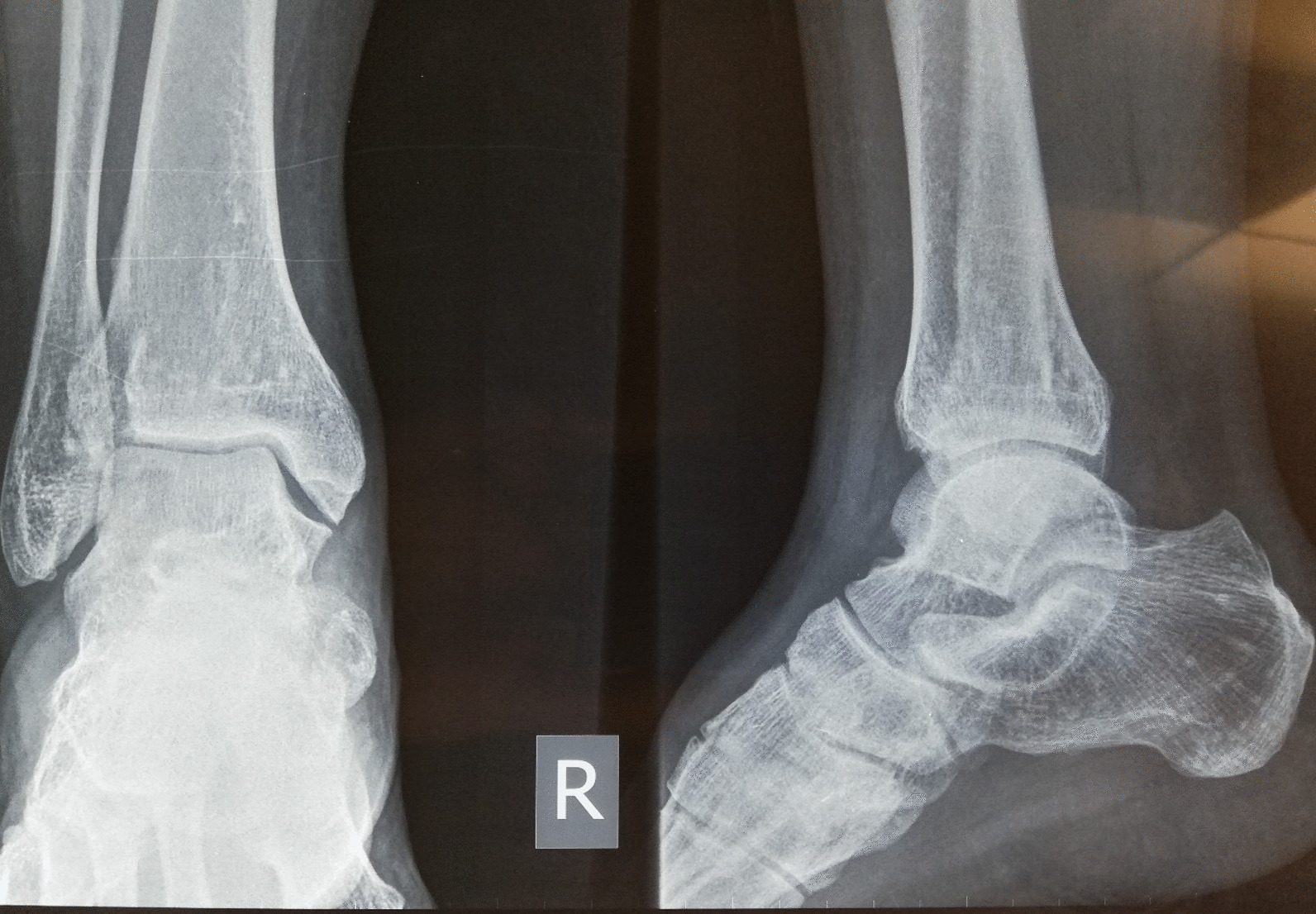



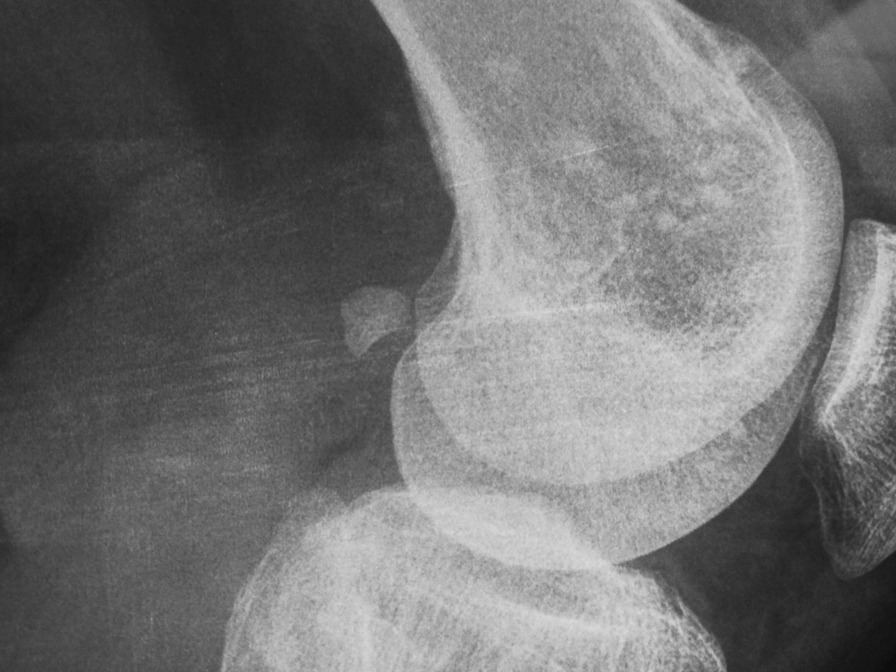



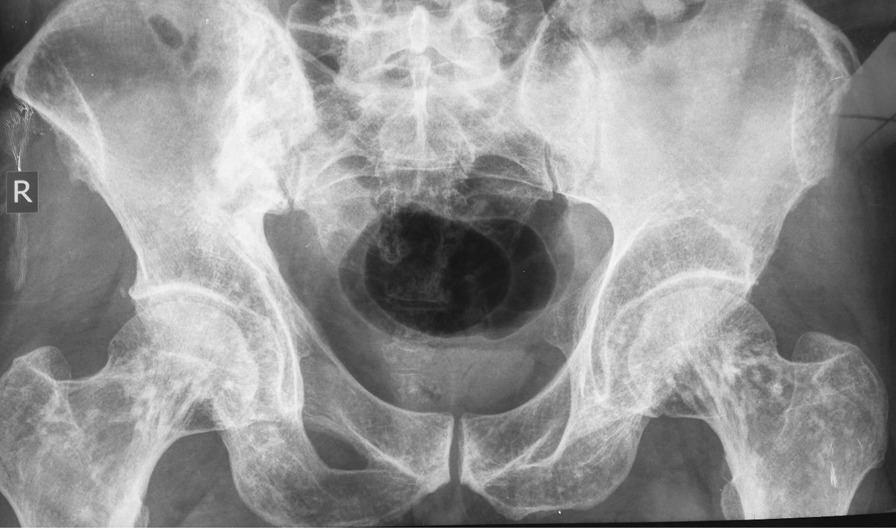



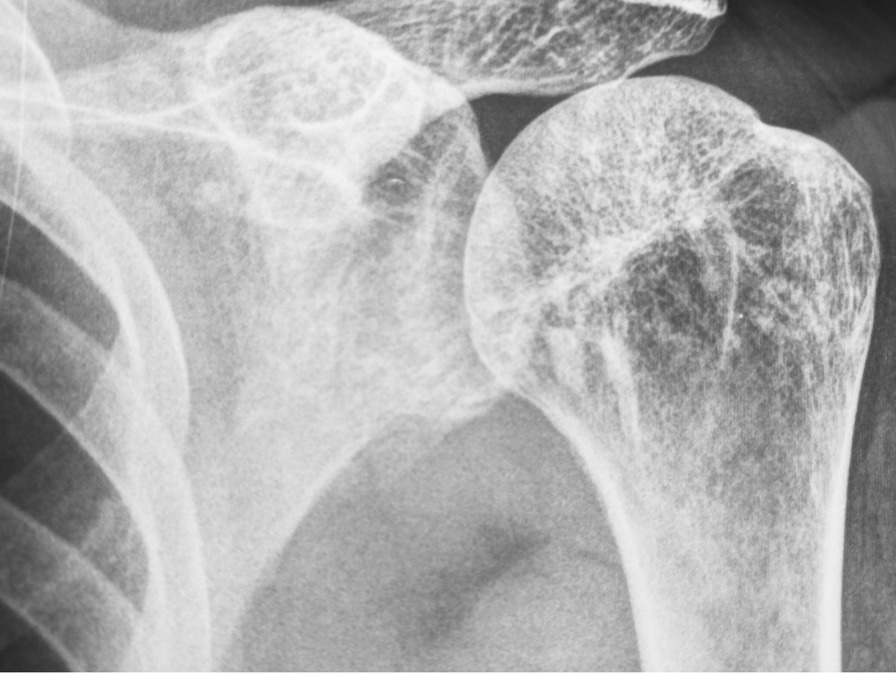



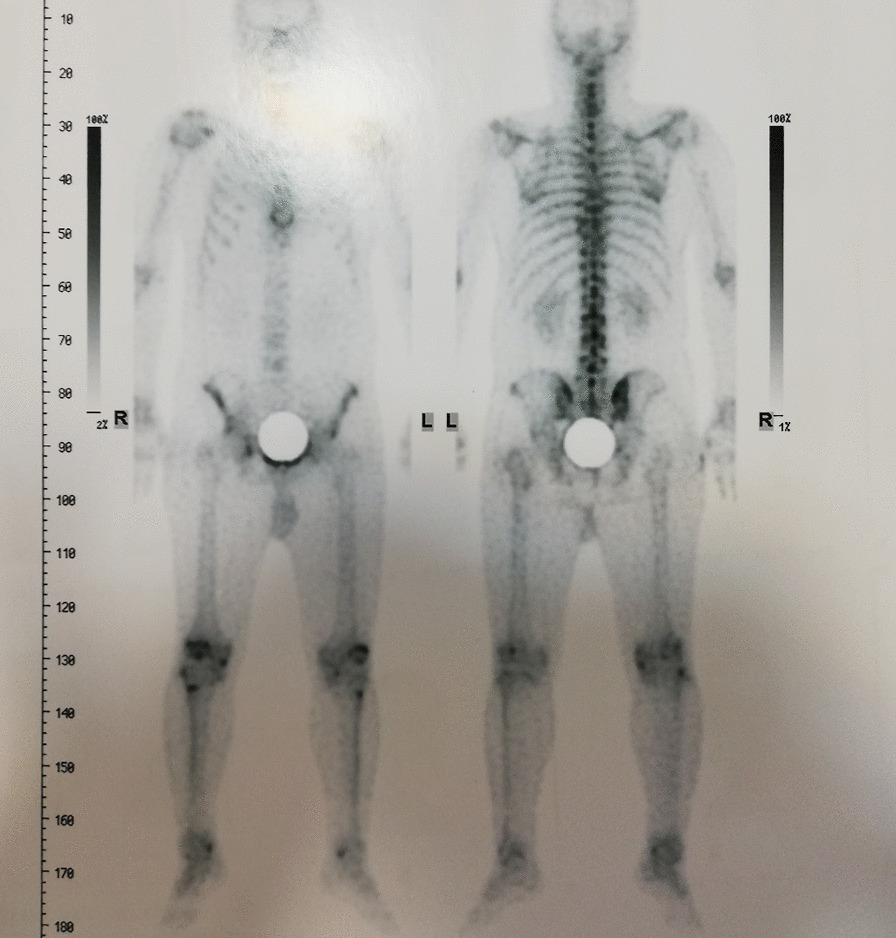



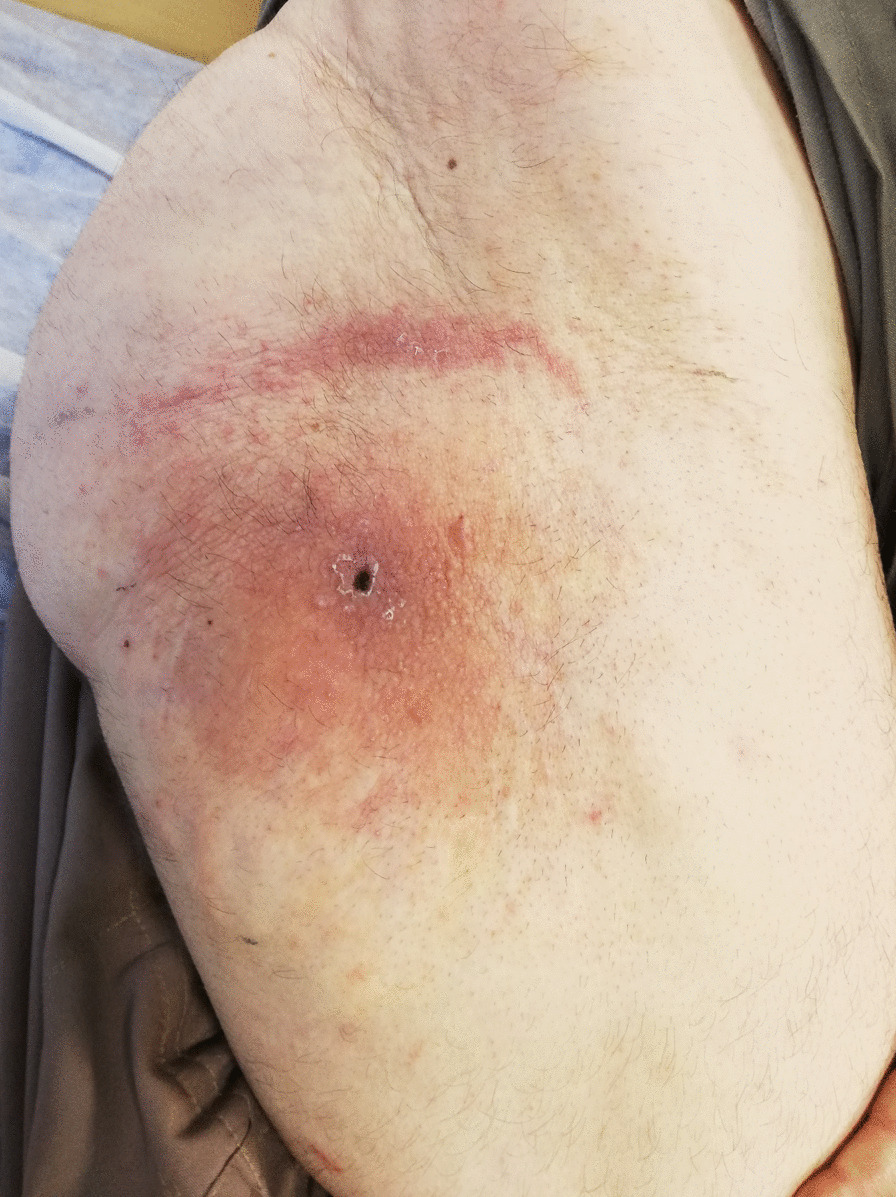



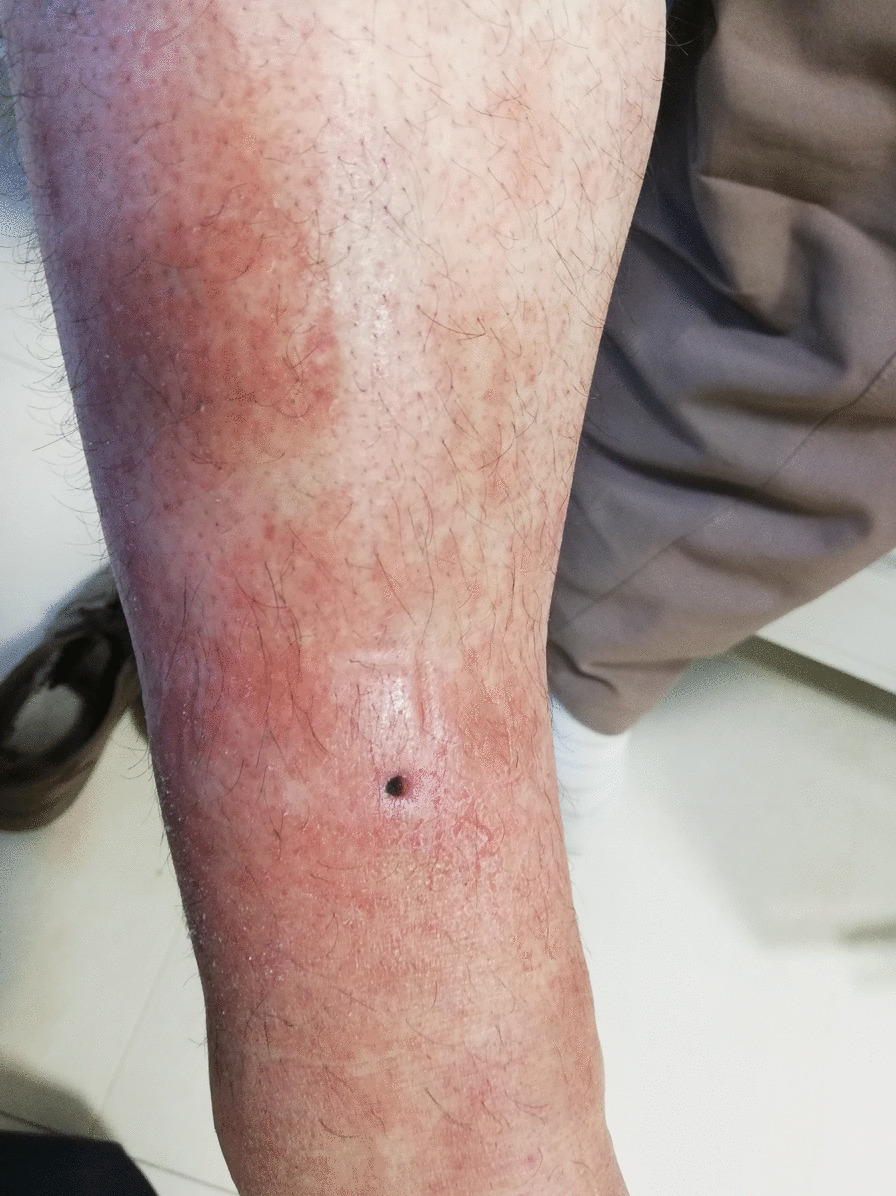


## Discussion

Some studies have reported a similar prevalence of the disease in men and women, while others have indicated a higher prevalence in men. This difference may be attributed to referral bias, as more men tend to visit the emergency department for traumatic accidents and injuries requiring X-rays. In reality, the incidence of the disease is similar in both genders [11]. There are reports of this syndrome coexisting with other conditions such as dwarfism, dystocia, coarctation of the aorta, double urethra, spinal canal stenosis, tuberous sclerosis, scleroderma, dystocia, precocious puberty, endocrine disorders (e.g., diabetes mellitus), fibromyalgia, facial anomalies, reactive arthritis, discoid lupus erythematosus (DLE), familial Mediterranean fever (FMF), and psoriatic arthritis [2, 3, 7, 8]. In bone histopathology, visible are lesions related to the lamellar osseous tissue containing the Haversian system and focal condensation of compact lamellar bone in the spongiest [3, 8]. The prevalence of bone involvement is as follows: phalanx (100%), carpal bone (97.4%), metacarpal (92.5%), toes (87.2%), metatarsus (84.4%), tarsus (84.6%), pelvis (74.4%), femur (74.4%), radius (66.7%), ulna (66.7%), sacrum (58.9%), humerus (28.2%), tibia (20.5%), and fibula (2.8%) [5, 8]. The most common anatomical locations of bone involvement are the epiphysis or metaphysis regions of the long bones [3]. Differential diagnoses of the disease include osteoblastic metastasis, primary bone tumor, mastocytosis, tuberous sclerosis, synovial chondromatosis, osteopathia striata, and melorheostosis [2, 3]. No specific point is observed in the bone scan with Technetium-99m, and the scans are often normal. However, the abnormality in the scan does not definitively rule out the diagnosis of osteopoikilosis [1, 2, 5, 7]. To date, four cases of osteopoikilosis with abnormal bone scans have been reported (2).

## Conclusions:

The patient is a 46-year-old man presenting with skin lesions and pain in the lower back, pelvis, neck, and limbs. The X-ray reveals shoulder, pelvis, knee, and ankle involvement. Spinal involvement is observed in the neck and lumbar region. The bone scan shows extensive enthesopathy in various regions, which is unique compared to previous cases and has not been reported thus far.

## Data Availability

All data generated or analyzed during this study were included in this article but the raw data are available from the corresponding author on reasonable request.
